# The role of resveratrol as a natural modulator in glia activation in experimental models of stroke

**Published:** 2020

**Authors:** Hamed Ghazavi, Shima Shirzad, Fatemeh Forouzanfar, Sajad Sahab Negah, Mona Riyahi Rad, Farzaneh Vafaee

**Affiliations:** 1 *Neuroscience Research Center, Mashhad University of Medical Sciences, Mashhad, Iran*; 2 *Department of Neuroscience, Faculty of Medicine, Mashhad University of Medical Sciences, Mashhad, Iran*; 3 *Shefa Neuroscience Research Center, Khatam-Alanbia Hospital, Tehran, Iran*; 4 *Department of Biology, Mashhad Branch, Islamic Azad University, Mashhad, Iran*

**Keywords:** Resveratrol, Stroke, Glia activation, Inflammation, Cytokines

## Abstract

**Objective:**

Stroke is one of the most important causes of death and disability in modern and developing societies. In a stroke, both the glial cells and neurons develop apoptosis due to decreased cellular access to glucose and oxygen. Resveratrol (3, 5, 4′-trihydroxy-trans-stilbene) as a herbal compound shows neuroprotective and glioprotective effects. This article reviews how resveratrol can alleviate symptoms after stroke to help neurons to survive by modulating some signaling pathways in glia.

**Materials and Methods:**

Various databases such as ISI Web of Knowledge, Scopus, Medline, PubMed, and Google Scholar, were searched from 2000 to February 2020 to gather the required articles using appropriate keywords.

**Results:**

Resveratrol enhances anti-inflammatory and decreases inflammatory cytokines by affecting the signaling pathways in microglia such as AMP-activated protein kinase (5' adenosine monophosphate-activated protein kinase, AMPK), SIRT1 (sirtuin 1) and SOCS1 (suppressor of cytokine signaling 1). Furthermore, through miR-155 overexpressing in microglia, resveratrol promotes M2 phenotype polarization. Resveratrol also increases AMPK and inhibits GSK-3β (glycogen synthase kinase *3* beta) activity in astrocytes, which release energy, makes ATP available to neurons and reduces reactive oxygen species (ROS). Besides, resveratrol increases oligodendrocyte survival, which can lead to maintaining post-stroke brain homeostasis.

**Conclusion:**

These results suggest that resveratrol can be considered a novel therapeutic agent for the reduction of stroke symptoms that can not only affect neuronal function but also play an important role in reducing neurotoxicity by altering glial activity and signaling.

## Introduction

Stroke is the third leading cause of mortality and one of the most important factors causing disability worldwide, which inflicts a great financial burden on both the individual and society (Towfighi and Saver, 2011 ▶). Cerebral ischemia is a neurological situation that results from a decrease in cerebral blood flow due to arterial occlusion. In experimental models of stroke (e. g. focal ischemia), a cerebral artery is artificially occluded and the insult results in the formation of successive areas where the blood flow changes gradually. Two principal metabolic conditions can be observed; the core, where the blood flow reduction is at maximum, and the outermost boundary (penumbra) where it is decreased in comparison to normal blood flow (Vafaee et al., 2014 ▶; Ghazavi et al., 2017 ▶; Pineda-Ramírez et al., 2018 ▶). Complicated cascades of events and neurovascular network dysfunction leading to severe brain damage, are induced by cerebral ischemia (Brouns and De Deyn, 2009 ▶). Various studies showed that in addition to neurons and vascular elements, glial cells play an essential role in the pathophysiology of stroke (Jayaraj et al., 2019 ▶). Various immune cells were recruited into the injured brain in the acute phase of inflammation (Anrather and Iadecola, 2016 ▶). This activation leads to release of higher levels of pro-inflammatory cytokines, which might last for weeks after cerebral ischemia (Liguz-Lecznar and Kossut, 2013 ▶). Resveratrol [(3, 5, 4′-trihydroxy-trans-stilbene) (RSV)] is a polyphenol and a natural plant antibiotic, found in grapes and peanuts that is widely known for its significant neuroprotective, anti-inflammatory, antioxidant, anticancer, and antiapoptotic properties (Harikumar and Aggarwal, 2008 ▶; Koushki et al., 2018 ▶). Importantly, RSV can cross through the blood-brain barrier to act as a powerful neuroprotective agent *in vivo* and *in vitro* (Bastianetto et al., 2015 ▶). RSV has also shown neuroprotective effects via ameliorating kainate-induced excitotoxicity and improves pathological and behavioral outcomes in various types of central nervous system (CNS) injuries such as stroke, traumatic brain injury (TBI), and spinal cord injury (SCI) (Zhang et al., 2010 ▶; Orsu et al., 2013 ▶; Yang et al., 2016 ▶; Lin et al., 2019 ▶). RSV has also gained attention for its anti-inflammatory properties (Das and Das, 2007 ▶). By inhibition of the transcriptional factors such as nuclear factor-kappaB (NF-κB), RSV can reduce the activation of immune cells and the subsequent synthesis and release of pro-inflammatory mediators (Das and Das, 2007 ▶). It was also shown that RSV modulated the production of interleukin 10 (IL-10) in the microglia and protected neurons and glia from oxidative stress-induced injury (Cianciulli et al., 2015 ▶). The anti-apoptotic effect of RSV has been shown at lower doses (2.5 or 5 mg/kg) and at a higher dose, it can act as a pro-apoptotic compound, inducing apoptosis in cancer cells (Mukherjee et al., 2010 ▶; Singh et al., 2013 ▶). It was demonstrated that RSV (30 mg/kg) could reduce brain damage in an ischemia-reperfusion model (Singh et al., 2013 ▶). Furthermore, it was shown that RSV has some antihypertensive effects (Theodotou et al., 2017 ▶) which can be helpful in stroke patients. It was reported that RSV does not have any side effects at short-term doses (1g/day), but side effects such as nausea, vomiting, and diarrhea may occur at doses of 2.5 g/day or more. It was also reported that there are not any major side effects in long-term clinical trials and RSV can be considered safe and tolerated at up to 5 g/day (Salehi et al., 2018 ▶). Taken together, RSV can play a role as a potential therapeutic agent in the treatment of cerebrovascular disease. This review discusses the role of RSV in glia activity and neurotoxicity following cerebral stroke.

## Materials and methods

Online literature resources were checked using different search engines such as ISI Web of Knowledge, Medline, PubMed, Scopus, and Google Scholar, from 2000 to February 2020 to identify articles about the contribution of RSV to glial activity after stroke. For this, the search terms “Resveratrol”, "RSV" OR "RSE" AND "Glia" OR "Astrocyte" OR "Microglia" OR "Oligodendrocyte" AND "AMPK" AND "SIRT1" OR " SOCS1" OR "SIRT1-SOCS1" AND "GSK-3β" AND "miR-155" OR "MicroRNA-155" AND " Nrf2" OR "HO-1" OR "Nrf2- HO-1" were used. 

## Results

Pro-inflammatory cytokines such as nitric oxide (NO), tumor necrosis factor-alpha (TNF-α), interleukin 1 beta (IL-1β), and interleukin 6 (IL-6), are secreted by activated glial cells leading to increased post-stroke inflammation (Leonoudakis et al., 2017 ▶). Various studies reported that RSV reduces the secretion of NO and TNF-α in the cell culture of microglia and astrocytes (Lorenz et al., 2003 ▶; Bi et al., 2005 ▶). Microglia and astrocytes are two of the most important cells that secrete pro-inflammatory cytokines, and RSV could modulate their inflammatory function and enhance neuron protection and recovery (Lu et al., 2010 ▶). Also, astrocytes and oligodendrocytes injury damages the myelin of neurons and the blood-brain barrier (Bonfanti et al., 2017 ▶; Wang et al., 2020 ▶), and RSV not only helps the neurons to survive but also improves neuronal function by supporting these glial cells (Rosa et al., 2018 ▶). Given the importance of glial cells' role in the pathophysiology of stroke, examples of RSV function on glial cells after stroke are described in Table 1. 

Moreover, according to Table 2, it was demonstrated that RSV plays an important role in the glial cell activity, such as decreasing inflammation, glioprotectective effects, and microglia polarity through affecting different signaling pathways such as activating glial AMP-activated protein kinase (AMPK), nuclear factor erythroid 2-related factor 2/ heme oxygenase 1 (Nrf2/HO-1) signaling, sirtuin 1 (SIRT1), and suppressor of cytokine signaling 1 (SOCS1) pathways, and inhibition glial microRNA-155 (miR-155), and glycogen synthase kinase-3β (GSK-3β), which will be discussed in detail later.

**RSV increases cell survival via glial AMP-activated protein kinase (AMPK) pathway**

AMPK as a highly conserved serine-threonine kinase through evolution, is found in most mammalian tissues, and is especially highly expressed in the brain and acts as a key sensor of brain energy balance and stress sensor/effector through astrocytes activity. It can be activated under different situations of heat shock, vigorous exercise and energy-deprived states such as cerebral ischemia (Schimmack et al., 2006 ▶; Li and McCullough, 2010 ▶; Mihaylova and Shaw, 2011 ▶). 

**Figure 1:**
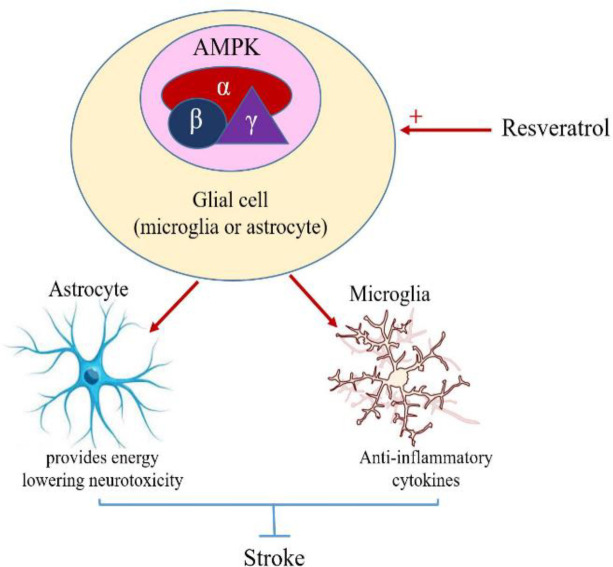
Rresveratrol increases the survival of neural cells by activating astrocytes and microglia AMPK pathway; AMPK activity triggers glycolysis and oxidation of fatty acids in astrocytes, which then provides the energy needed for the damaged neurons and help them to survive. AMPK can inhibit the secretion of inflammatory factors and stimulate anti-inflammatory factors from microglia

RSV has shown a neuroprotective effect via upregulating AMPK signaling pathway after stroke (Lin et al., 2019 ▶). It was also reported that RSV in a Parkinson's model, increased neuronal viability by activating the astroglia AMPK signaling pathway (Park et al., 2019b ▶). AMPK plays a vital role in maintaining energy for cells and keeping the brain homeostasis (Carling, 2017 ▶). RSV by increasing AMPK activity provides the energy needed for neurons to survive in the absence of oxygen and glucose (Lin et al., 2019 ▶). Also, in severe ischemia due to damage to astrocytes, decreasing glycogen stores and absence of the key enzymes to produce ATP via glycolysis, and inefficient oxidation of fatty acids in neurons, there is a propagating acidosis and metabolic failure (Schimmack et al., 2006 ▶; Mihaylova and Shaw, 2011 ▶). As shown in Figure 1, the upregulation of AMPK by RSV can activate glycolysis and fatty acids oxidation to form ketones in astrocytes, besides storing some glycogen, provides a short-term energy supply for ischemic neurons (Favero and Mandell, 2007 ▶; Li and McCullough, 2010 ▶; Pineda-Ramírez et al., 2018 ▶). Phosphofructokinase 2 (PFK2) is an enzyme responsible for glycolysis in cells. 

**Table 1:** Effects of resveratrol on glial cell in experimental models of stroke

**Resveratrol treatment**	**Model**	**Glial cell type**	**Results**	**Ref.**
100 mg/kg i.p. once per day for 14 days	Intracerebral hemorrhage	Microglia	↓Secondary brain injury ↑Motor abilities ↓Neural damage ↓Activation of microglia	(Cai et al., 2018 ▶)
50 nmol/μL at L4–6 segments of the spinal cord	Spinal cord ischemia/reperfusion	Astrocytes Microglial	↓Inflammation	(Li et al., 2014 ▶)
30 mg/kg i.p. at 24 h after ischemia	Global cerebral ischemic injury	Astrocytes Microglial	↓Cerebral ischemic injury	(Wang et al.,2002 ▶)
25 μmol/L (culture medium)	Rodent astrocyte cultures and an in vivo stroke model	Astrocytes	↑Mitochondrial function	(Narayanan et al., 2015 ▶)
20 mg/Kg i. p.	Hypoxic–ischemic model	Astrocytes	↓Infarct volume ↑Mitochondrial function ↓ROS ↓Neuronal loss	(Arteaga et al., 2015 ▶)
20 mg/kg i. p; 10 min before hypoxia	Hypoxic–ischemic model	Astrocytes Microglial	↓TNF-α, COX2, caspase 3 No differences were observed in SOD2 expression	(Revuelta et al., 2017 ▶)
1, 10mg/kg i.p for 21 days	Forebrain global ischemia	Astrocytes Microglial	↓GLT-1 expression ↑CA1 neuronal protection	(Girbovan and Plamondon, 2015 ▶)
20 mg/kg, 10 min before hypoxia	Hypoxic ischemic	Astrocytes Oligodendrocytes	↑Body and brain weight ↓ROS	(Revuelta et al. 2016 ▶)
30, 60, 90 mg/kg i.p. at 2 and 12 h post-injury 1, 10, 20 µM (culture medium)	Subarachnoid hemorrhage and in vitro experiment in primary cultured cortical neurons	Microglial	↓Inflammation ↓Brain edema ↓Neurobehavioral impairment ↓Apoptosis ↓Neuronal degeneration	(Zhang et al., 2017b ▶)
60 mg/kg i.p., 1 h after SAH	Subarachnoid hemorrhage	Astrocytes Microglial	↓Inflammation ↓Apoptosis	(Zhao et al., 2017 ▶)
1, 2.5, or 5 mg/kg, i.v, 3 or 6 h after MCAO	Middle cerebral artery occlusion model	Microglia	↓IL-1β ↓TNF-α ↓Microglial activation ↓ROS	(Shin et al., 2010 ▶)
60 mg/kg at 2 and 12 h post-injury	Subarachnoid hemorrhage	Microglia	↓Microglia activation ↓Inflammatory cytokines ↓Apoptosis ↓Brain edema ↓Neurological deficit score ↓NF-κB	(Zhang et al., 2016a ▶)
20, 50 mg/kg i.p, daily pre-ischemic injections starting on day 7	Focal cerebral ischemia	Astrocytes Microglia	↓Infarct volume ↑Blood-brain-barrier integrity ↓Bax, caspase-3 ↑Angiogenesis and neurogenesis ↑GDNF and VEGF	(Hermann et al., 2015 ▶)
100 mg/kg, i.p., injected three times at 0 h, 8 h and 18 h after hypoxic-ischemic	Neonatal hypoxic-ischemic	Microglia	↓Inflammation ↓Microglia activation ↓Apoptosis ↓Bax ↓Bcl-2 ↓Caspase3	(Pan et al., 2016 ▶)
30 mg/kg, i.p., every day for 7 days before ischemia	Global ischemia	Astrocytes Microglia	↓Astroglial and microglial activation ↓COX2 and iNOS production ↓Inflammation	(Simão et al., 2012 ▶)
100 mg/kg i.p., injected at 0, 8 and 18 h after cerebral ischemia	Middle cerebral artery occlusion	Microglia	↓MiR-155 expression ↑M2 polarization of microglia ↓Neuroinflammation	(Ma et al., 2019 ▶)
5, 10, 20, 40 mg/ kg, i.p., administered from day 1 after surgery till 27 days	Intracerebral hemorrhage	Microglia	↑Neurological scoring tests ↑Anti-inflammatory and antioxidant factors	(Singh et al., 2017 ▶)
10 mg/kg i.p for 20 days	Ischemia/reperfusion	Microglia	↓Inflammation ↓Ionized calcium binding adaptor molecule 1 ↓Neuronal apoptosis	(Zhao et al., 2019 ▶)

Bax: Bcl-2-associated x protein; Bcl-2: B-cell lymphoma 2; COX2: Cyclooxygenase 2; SOD2: Superoxide dismutase 2; DMSO: Dimethyl sulfoxide; GDNF: Glial cell-derived neurotrophic factor; GLT-1: Glutamate transporter 1; HI: hypoxic-ischemic; i.p: Intraperitoneal; ICH: Intracerebral hemorrhage; IL-1β: Interleukin 1 beta; iNOS: Inducible nitric oxide synthase; MCAO: Middle cerebral artery occlusion; NF-κB: Nuclear factor kappa-light-chain-enhancer of activated B cells; ROS: Reactive oxygen species; SAH: Subarachnoid hemorrhage; TNF-α: Tumor necrosis factor alpha; VEGF: Vascular endothelial growth factor.

Post-inflammation increasing of NO has a cytoprotective effect due to stimulating glycolysis via activation of AMPK and increasing the PFK2 concentrations (Rider et al., 2004 ▶). Also, it was shown that mice deficient in AMPK in astrocytes had a worse functional recovery after stroke (Favero and Mandell, 2007 ▶). It was also suggested that RSV enhances PFK2 in astrocytes which can be activated by AMPK, leading to enhanced glycolysis and increased glycolytic products such as pyruvate, lactate, and ATP (Favero and Mandell, 2007 ▶; Li and McCullough, 2010 ▶; Pineda-Ramírez et al., 2018 ▶). It is important to bear in mind that progressive lactic acidosis leads to neuronal death after periods of prolonged injury such as that observed in stroke. This emphasizes the importance of examining the effect of loss of AMPK, selectively in neurons or astrocytes following stroke (Favero and Mandell, 2007 ▶; Li and McCullough, 2010 ▶). Compelling evidence also showed that RSV by AMPK activation in astrocytes, may protect neurons due to lowering calcium influx, and reducing excitotoxicity and likely diminishes cerebral ischemic injury (Girbovan and Plamondon, 2015 ▶; Maixner et al., 2015 ▶; Pineda-Ramírez et al., 2018 ▶). As we know, overexpression of glutamate after stroke causes neurotoxicity, and several studies showed that calcium secretion from astrocytes due to extracellular glutamate stimulation, is one of the important mechanisms involved in neurotoxicity (Mahmoud et al., 2019 ▶). RSV-activated AMPK can decrease the calcium influx and neurotoxicity by increasing the initialization of glial glutamate transporter-1(GLUT-1), which is responsible for transferring glutamate into astrocytes (Zhang et al., 2015 ▶; Girbovan and Plamondon, 2015 ▶; Maixner et al., 2015 ▶; León et al., 2017 ▶). Microglia are the remnants of immune cells that increase inflammation and ROS by secreting cytotoxic cytokines after a stroke; That is why they are an important therapeutic target in stroke (Chen et al., 2014b ▶). Based on Table 1, we found many studies indicating that the effects of RSV on microglia could reduce the inflammation and improve the symptoms of a stroke. RSV can activate AMPK in the microglia, which participates in the treatment of tumor cell implantation-induced neuroinflammation. It activates AMPK to hinder microglial activation and reverse the production of TNF-α and IL-1β (Figure 1). These results well documented the important role of AMPK in neuroglial cells (Song et al., 2015 ▶; Yang et al., 2016 ▶). It was also demonstrated that AMPK activation could inhibit microglial activation and relieve microglia-mediated neuroinflammation, thus alleviate trigeminal neuralgia (Wight et al., 2012 ▶; Zhang et al., 2013 ▶; Yang et al., 2016 ▶). NF‐κB activation, induced by upstream signals from diverse immune receptors, can be inhibited by RSV (Yuan et al., 2013 ▶; Liu et al., 2014 ▶). Activation of NF‐κB may play a pivotal role in pro‐inflammatory cytokines production in response to microglia activation. NF-κB subunits are not direct phosphorylation targets of AMPK, and several downstream targets of AMPK mediated the inhibition of NF-κB signaling (Salminen et al., 2011 ▶). NF‐κB activation, which was implicated as the first signal priming the synthesis of inflammatory cytokines in microglia, can be attenuated by RSV (Han et al., 2014 ▶).

**RSV suppresses microglial cells through activating sirtuin 1 (SIRT1) and suppressor of cytokine signaling 1 (SOCS1) pathways**

SIRT1, as a member of the sirtuin family, has a pivotal role in key cellular processes and could modulate a variety of biological functions, such as oxidative stress, immune response, mitochondrial biogenesis, and apoptosis/autophagy. It has gained attention that Sirt1 has a neuroprotective effect against cerebral ischemia/reperfusion (I/R) injury (Chen et al., 2005 ▶; Rovillain et al., 2011 ▶; Cho et al., 2015 ▶; Zhang et al., 2016b ▶; Yan et al., 2019 ▶). 

**Table 2:** Effects of resveratrol on glial cell signaling

**Resveratrol treatment**	**Model**	**Glia type**	**Signaling**	**Main finding**	**Ref**
5, 25, and 125 μM	Microglial and astrocyte cell line	Astrocyte Microglia	AMPK	↓Inflammation	(Yang et al., 2016 ▶)
125 μM					(Song et al., 2015 ▶)
40, 80 or 160 mg/kg	Morphine-induced microglial activation	Microglia		↓Microglial activation	(Han et al., 2014 ▶)
10 or 25 μM	Microglia cell lines treated with LPS	Microglia	SIRT1	↓Proinflammatory cytokines from microglial activation	(Ye et al., 2013 ▶)
10 mg/ kg/day	Postoperative cognitive dysfunction			↓Inflammation ↓NF-κB and IL-6 expression ↑Spatial memory	(Yan et al., 2019 ▶)
15 or 30 μM	Microglia cell lines treated with LPS			↓Inflammation ↓MicroRNA activity related to inflammation	(Li et al., 2015 ▶)
30-50 μM	Microglia cell culture from Aβ-(1-42)-induced Alzheimer’s disease			↓Microglial NF-κB signaling	(Chen et al., 2005 ▶)
5, 10 or 20 mg/kg	LPS-induced spatial memory impairment	Astrocyte Microglia		↓Inflammation ↑Synaptophysin expression ↑Spatial memory	(Chen et al., 2017b ▶)
5, 10, 25, and 50 μM	Microglia cell lines treated with LPS	Microglia	SIRT1-SOCS1	↓Microglial activation ↓Inflammation	(Zhang et al., 2017a ▶)
10 μM	Microglia cell lines treated with LPS	Microglia	SOCS1	↓Microglial activation ↓Inflammation	(Dragone et al., 2014 ▶)
10 to 100 mg/kg	MPTP mouse model of Parkinson's-like disease			↓Inflammation Protects dopaminergic neurons	(Lofrumento et al., 2014 ▶)
1, 5, 10, and 20 µM	LPS-stimulated microglia			↓Inflammation Inhibiting miR-155	(Ma et al., 2017 ▶)
30 mg/kg	LPS-induced neuroinflammation	Microglia	M1 to M2	↓Inflammation	(Yang et al., 2017 ▶)
100 mg/kg	Middle cerebral artery occlusion			↓Inflammation inhibiting miR-155	(Ma et al., 2019 ▶)
100 mg/kg	Traumatic brain injury	Astrocyte	GSK-3β	↑Cell survival ↓Autophagy and apoptosis	(Lin et al., 2014 ▶)
10 μM	LPS-mediated cytotoxicity	Oligodendrocyte	Nrf2/HO-1	↓Inflammation ↑Oligodendrocyte function ↓NFκB	(Rosa et al., 2018 ▶)

AMPK: AMP-activated protein kinase; GSK-3β: Glycogen synthase kinase 3 beta; LPS: lipopolysaccharide; miR-155: MicroRNA-155; MPTP: 1-methyl-4-phenyl-1,2,3,6-tetrahydropyridine; NF-κB: Nuclear factor kappa-light-chain-enhancer of activated B cells; Nrf2/HO-1: Nuclear factor erythroid 2–related factor 2/ heme-oxygenase-1; SIRT1: Sirtuin 1; SOCS1: Suppressor of cytokine signaling 1.

**Figure 2:**
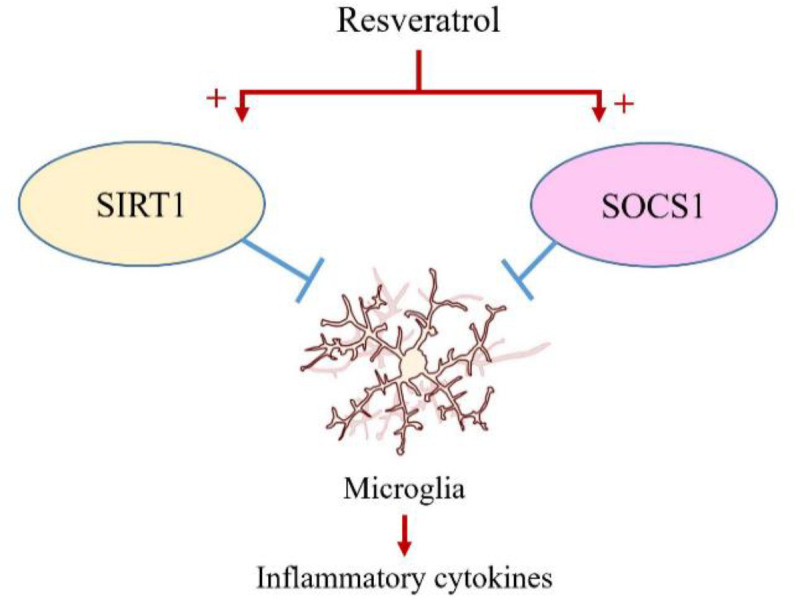
Inhibition of microglia inflammatory cytokines secretion; Following the stroke, microglial activity is increased, leading to the production of inflammatory cytokines. On the other hand, SIRT1 and SOCS1 function decrease after stroke. Resveratrol acts as a SIRT1 agonist and increases SOCS1 expression, thereby inhibiting the secretion of inflammatory cytokines from microglia

According to Table 1, RSV works as an anti-inflammatory agent and improves cerebral stroke outcomes. Pieces of evidence showed that the anti-inflammatory effect of RSV is due to its function as an activator of SIRT1 and inhibitor of the microglial activation (Chen et al., 2014b ▶; Chen et al., 2017b ▶). Recently, some studies demonstrated that SIRT1 prevents neuronal damage and long-term neurologic dysfunction. It was demonstrated that SIRT1 promoted recovery of neuron function and enhanced neuronal survival via modulating macrophage/microglia polarization (Jęśko et al., 2017 ▶; Chen et al., 2017a ▶). In addition, SIRT1 deacetylates intracellular targets such as signaling molecules and transcription factors in microglia that is followed by a reduction in inflammatory cytokines. Taken together, RSV by activating SIRT1 could protect against cellular senescence, ROS, and inflammation. It was shown that in primary cortical cultures, overexpression of SIRT1 in microglia has a protective effect against Aβ toxicity, probably by inhibiting NF-κB signaling (Chen et al., 2005 ▶; Rovillain et al., 2011 ▶; Chen et al., 2014a ▶; Cho et al., 2015 ▶; Zhang et al., 2016b ▶; Yan et al., 2019 ▶). RSV could improve cognitive dysfunction in Alzheimer's disease by activating microglial SIRT1 (Quadros Gomes et al., 2018 ▶). Overexertion of SIRT1 by RSV inhibits microRNAs related to inflammatory pathways especially in microglia (Li et al., 2015 ▶). Some evidence suggests that microglial cells contribute to post-stroke inflammation and regulation of microglia activation is a crucial key for neuron functional recovery. It was also reported that SIRT1 activators such as SRT1720 and RSV could improve the recovery of neuron function by modulating microglia activation (Meng et al., 2015 ▶; Chen et al., 2017b ▶; Lu et al., 2019 ▶) (Figure 2). It was also indicated that anti-inflammatory effects in microglia and macrophages can be induced by the overexpression of SOCS proteins. The SOCS proteins have gained attention due to their potential use as immunomodulators in diseases. These proteins, especially SOCS1 and SOCS3, are expressed by immune cells of the central nervous system (CNS) including microglia and were shown to have the potential to impact immune processes within the CNS, such as inflammatory cytokine and chemokine production (Baker et al., 2009 ▶). RSV can inhibit lipopolysaccharides (LPS)-induced microglial activation via the SIRT1 and SOCS1 pathways which may be associated with the modulation of microglial activated states (Zhang et al., 2017a ▶). On the other hand, RSV increases the SOCS1 protein expression, which reveals that the SOCS1 protein might be involved in the anti-inflammatory effects of RSV (Nosho et al., 2009 ▶; Meng et al., 2015 ▶; Zhang et al., 2017a ▶) (Figure 2). In the study conducted by Dragone et al, it was shown that RSV has anti-inflammatory effects in murine microglial cells stimulated by LPS, via up-regulating SOCS1 expression (Dragone et al., 2014 ▶). In other studies, RSV treatment significantly reduced glial activation and decreased the levels of IL-1β, IL-6, and TNF-α in the brain tissue through upregulating the SOCS1 protein expression in a 1-methyl-4-phenyl-1, 2, 3, 6-tetrahydropyridine- (MPTP)-induced Parkinson's disease (PD) mouse model (Lofrumento et al., 2014 ▶) and decreased the immune response of LPS-stimulated macrophages via the SOCS1 pathway (Ma et al., 2017 ▶). 

AMPK: AMP-activated protein kinase

**RSV enhances the polarization of microglia to M2-type through inhibition of miR-155**

Microglia as immune surveilling cells of the CNS, play an important role in maintaining homeostasis in the healthy brain, but when they are exposed to injury or infection, they become activated and secrete pro-inflammatory and neurotoxic mediators. Since sustained production of these factors can lead to worsening of neuronal damage, inhibition of microglia-mediated neuroinflammation has gained attention as a promising therapeutic target for neurological disorders (Das and Das, 2007 ▶; Vafaee et al., 2018 ▶). According to the predominance of secreted cytokines, activated microglia are classified into the M1 phenotype (pro-inflammatory) and M2 phenotype (anti-inflammatory). Based on what we know, it could be deduced that the inhibition of M1 polarization and the promotion of M2 polarization of microglia might be a novel therapeutic approach in inflammation-related diseases (Hu et al., 2012 ▶; Xu et al., 2015 ▶).

SIRT1: Sirtuin 1; SOCS 1: suppressor of cytokine signaling 1.

**Figure 3:**
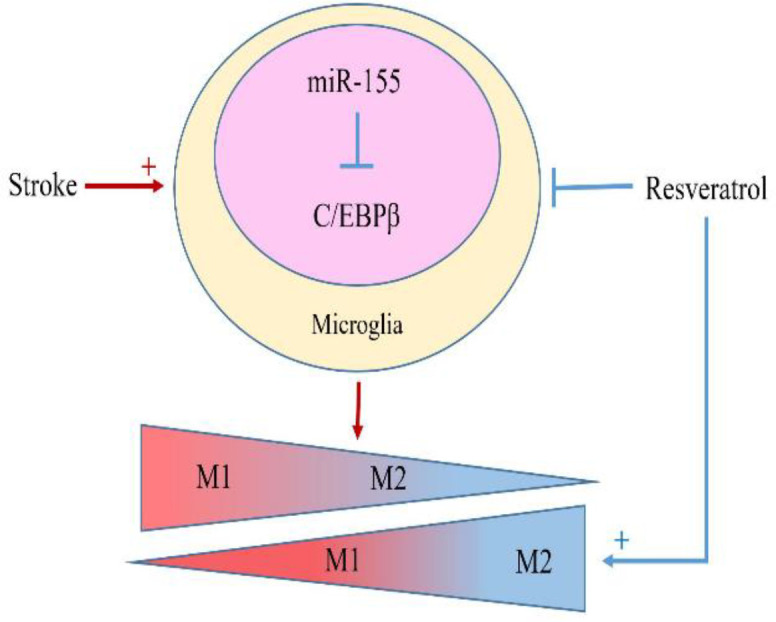
Resveratrol activates the M2 Microglia; Stroke-induced brain injury causes the upregulation of miR-155 in microglia, which leads to differentiation of microglia into the M1 phenotype through inhibiting C/EBPβ. Resveratrol by down-regulation of miR-155 can lead to the differentiation of microglia into the M2 phenotype

 Several studies demonstrated that RSV obviously reduced neuroinflammation after cerebral ischemia through inhibiting M1 polarization and promoting M2 polarization of microglia. It was indicated that RSV can promote M2 polarization of microglia and reduce neuro-inflammation by inhibiting miR-155 (Zhang et al., 2018 ▶; Ma et al., 2019 ▶). MiR-155 is upregulated after cerebral ischemia and was identified and characterized as a component of microglia response to different types of inflammatory mediators, such as LPS, interferon- beta (IFN-β) and TNF-α and can be down-regulated by RSV (Cardoso et al., 2012 ▶). Furthermore, the inhibition of miR-155 by RSV attenuates brain damage and further improves the neurological function recovery post-ischemia (Wen et al., 2015 ▶; Caballero-Garrido et al., 2015 ▶). Similarly, the down-regulation of miR-155 alleviates microglia-mediated neuron toxicity after SCI (Gaudet et al., 2016 ▶) (Figure 3). 

C/EBPβ; transcription factor CCAAT/enhancer-binding protein beta.

RSV activates the transcription factor CCAAT/enhancer-binding protein beta (C/EBPβ) by suppressing miR-155. C/EBPβ as a regulator of the expression of key genes in inflammation (Ejarque-Ortiz et al., 2007 ▶) is involved in the process of microglia polarization. It was also shown that microglia differentiation is defective in C/EBPβ-deficient mice (Shen et al., 2008 ▶; Schmeier et al., 2009 ▶). Through the conserved miR-155 binding site in the 3’UTR of C/EBPβ transcripts, miR-155 can modulate microglia polarization by inhibition of C/EBPβ expression at the posttranslational level (He et al., 2009 ▶; Zhang et al., 2018 ▶). It was also indicated that miR-155 might be an important therapeutic target in the CNS injury. RSV through affecting miR-155, can be involved in the process of modulating microglia polarization in the favor of anti-inflammatory phenotype.

**RSV protects astrocytes by inhibiting glycogen synthase kinase-3β (GSK-3β) activity**

**Figure 4:**
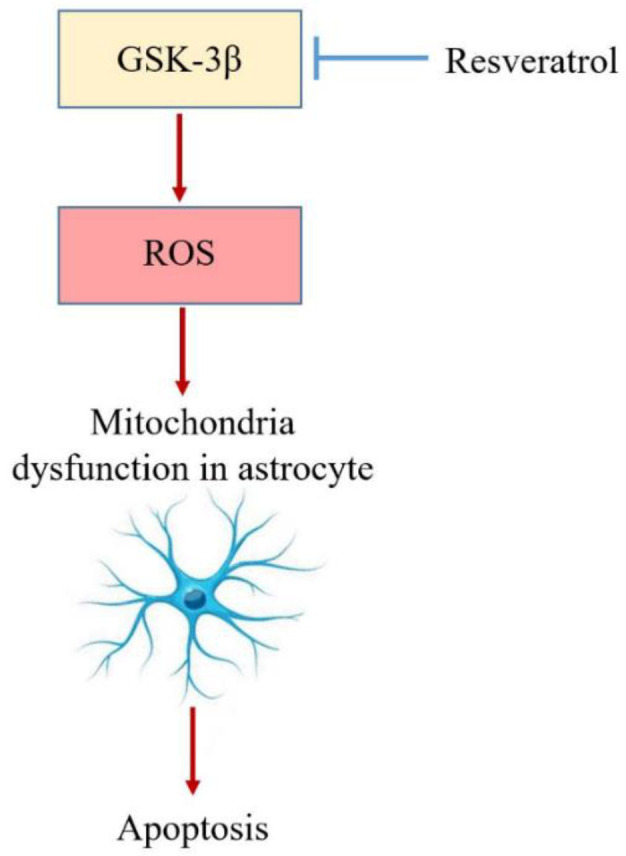
Protective role of resveratrol in astrocytes; increased GSK-3β activity in neurons increases ROS, leading to mitochondrial damage in astrocytes and activating their apoptotic pathway. Resveratrol inhibits astrocyte death by blocking the GSK-3β pathway

GSK-3: Glycogen synthase kinase 3; ROS: Reactive oxygen species.

GSK-3 as a serine/threonine kinase was named for its ability to phosphorylate and to inactivate glycogen synthase, an important regulatory enzyme in the synthesis of glycogen. It is now widely known that GSK3 has a key role in diverse signaling pathways involved in the regulation of cell fate, protein synthesis, glycogen metabolism, transformation, cell mobility, proliferation, and survival. GSK3α (51 kDa) and GSK3β (47 kDa) which are not functionally identical, are two highly homologous forms of GSK3 in mammals and are encoded by distinct genes (Jope et al., 2007 ▶; Beurel et al., 2015 ▶). GSK3β is particularly abundant in the CNS and is highly expressed in both neurons and glial cells (Luo, 2012 ▶). Previous studies demonstrated that RSV can reduce the area of tissue lesions in rats with TBI and improves neuronal function after SCI due to the anti-oxidative effect via suppressing GSK3β activity (Petit-Paitel et al., 2009; Lin et al., 2014 ▶). Several studies indicated that GSK3β plays an important role in neuronal development, controls neural progenitor homeostasis and neurogenesis. In this way, changes in local and global GSK3β activity has critical effects on axon/dendritic outgrowth and specification as well as neuronal migration (Yoshimura et al., 2005 ▶; Hur and Zhou, 2010 ▶; Kim and Snider, 2011 ▶; Jung et al., 2016 ▶). Up-regulation of GSK-3β activity after stroke can result in cell death and aberrant neuronal migration in primary neuronal populations. Studies reported that direct overexpression of GSK-3β as a major contributor to the control of cell fate within the central nervous system, can induce apoptosis in neuronal cells and astrocytes in culture, and this apoptotic response can be improved by specific inhibitors of GSK-3β (Sanchez et al., 2003 ▶; Koistinaho et al., 2011 ▶). It was reported that RSV regulates GSK-3β in neuroinflammation by affecting the astrocytes (McCubrey et al., 2017 ▶; Jhang et al., 2017 ▶; Park et al., 2019a ▶). Astrocytes as the most abundant glial cells in the CNS have important activities including transporting nutrients, holding neurons, and participating in neurotransmission. Under the condition of brain injury, other functions of astrocytes, such as uptake and release of glutamate, and release of substrates for neuronal energy metabolism have a strong impression on neuronal survival (Chen and Swanson, 2003 ▶; Burda et al., 2016 ▶). Normal astrocytes show apoptotic and autophagic cell death due to glutamate-induced ROS bursts which activate GSK-3β (Sanchez et al., 2003 ▶; Lin et al., 2014 ▶). Glutamate is the major excitatory neurotransmitter found in neuronal and glial cells of the mammalian CNS. Glutamate-induced astrocytes and neuronal apoptosis may be due to calcium overload and ROS generation. Moreover, due to the effects of astrocytes on neurogenesis and synaptic reorganization, the death or survival of them may affect the ultimate clinical outcome and rehabilitation (Chen and Swanson, 2003 ▶; Leon et al., 2009 ▶). GSK-3β may induce apoptosis through mitochondrial dysfunction. Conversely, inhibition of GSK-3β by RSV may protect against apoptosis and increase cell viability via suppressing ROS generation and GSK-3β activation. ROS activated GSK-3β causes mitochondrial dysfunction, which can result in cytotoxicity in astrocytes (Lin et al., 2014 ▶). It was noted that RSV can suppress the production of ROS and thereby reduces astrocytes' death (Figure 4). 

**RSV protects oligodendrocytes via enhancing** **nuclear factor erythroid 2-related factor 2/ heme oxygenase 1 (Nrf2/HO-1) signaling pathway**

**Figure 5:**
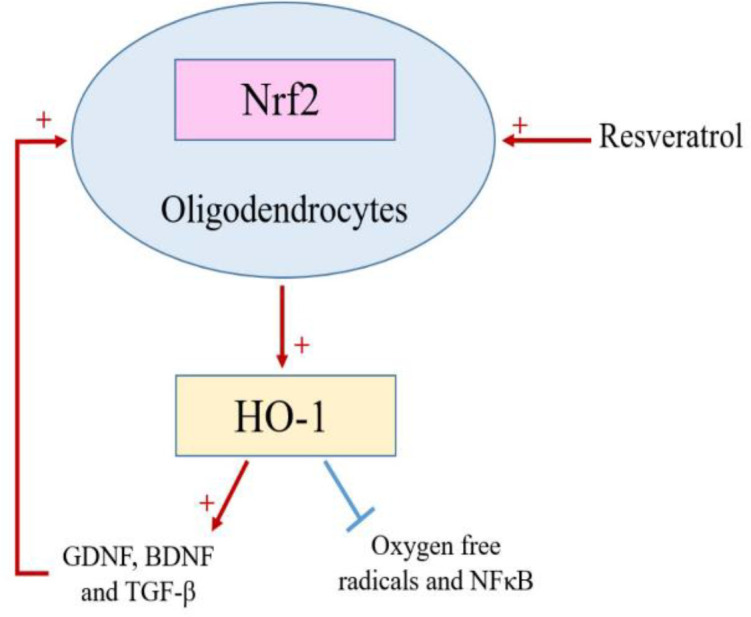
The role of resveratrol as an oligoprotective agent; Resveratrol activates the Nrf2/ HO-1 signaling pathway, resulting in a decrease in oxygen free radicals and NFκB inflammatory factor followed by increased GDNF, BDNF and TGF-β resulting in increased oligodendrocyte viability

Nrf2: Nuclear factor erythroid 2-related factor 2; HO-1: Heme oxygenase 1.

Oligodendrocytes as the myelin-forming glial cells in the CNS are vulnerable to damage in a variety of neurologic diseases. There is an increasing awareness that oligodendrocytes are targets of injury in acute ischemia. Similar to neurons, oligodendrocytes are highly sensitive to injury caused by oxidative stress, trophic factor deprivation, excitatory amino acids, and activation of apoptotic pathways (Dewar et al., 2003 ▶). Evidence indicated that RSV has oligoprotective effects and prevents cytotoxicity and oxidative stress through the oligodendrocyte Nrf2/HO-1 signaling pathway (Wang and Dore, 2007 ▶; Gao et al., 2018 ▶; Rosa et al., 2018 ▶) (Figure 5). 

 Several lines of evidence have highlighted that HO-1 expression is correlated with neuronal and oligodendrocytes damage especially in stroke. HO-1 up-regulation is not only recognized as a key mechanism of cell and glia adaptation to stress, but also under the control of different transcription factors concerning a prominent role played by Nrf2, is crucial in the response of the nervous/glia system to damage (Nitti et al., 2018 ▶; Liu et al., 2019 ▶). It was also demonstrated that regarding HO-1 as a therapeutic target in the CNS, these observations reinforce the oligoprotective effect of RSV, which may potentially be used in brain pathological conditions (Wang and Dore, 2007 ▶; Gao et al., 2018 ▶; Rosa et al., 2018 ▶) (Figure 5). Also, excessive microglial activation following cerebral stroke is toxic both to neurons and glial cells, such as oligodendrocytes. It was shown that reactive microglia in lesions, cause injuries to oligodendrocytes, leading to myelin defects by excessive production of oxidative stress and cytokines (Li et al., 2017 ▶). Changes in oligodendroglia functionality may compromise the CNS homeostasis and contribute to the pathogenesis of several neurological diseases such as stroke. Previous studies reported the crosstalk between oligodendrocytes and other cells such as neurons, astrocytes, and cerebral endothelium as an important function beyond myelination. Such crosstalk appears to involve the signaling of important trophic factors such as transforming growth factor β (TGF-β), brain-derived neurotrophic factor (BDNF), and glial cell-derived neurotrophic factor (GDNF) and RSV can reestablish the values of their release near control conditions (Dewar et al., 2003 ▶; Shen et al., 2008 ▶; Rosa et al., 2018 ▶). TGFβ signaling has an important role in several biological processes, including development, immune responses, migration, proliferation, and differentiation of OPC (Oligodendrocyte progenitor cells) (Palazuelos et al., 2014 ▶). Moreover, studies reported that in pathological conditions associated with neuroinflammation, such as cerebral stroke, Alzheimer's disease and amyotrophic lateral sclerosis (ALS), TGF-β release is increased (Wyss-Coray et al., 2000 ▶; Endo et al., 2015 ▶) and RSV by affecting oligodendrocytes, is able to prevent these changes. Furthermore, BDNF and GDNF secretion is decreased by inflammation, which can be prevented by RSV (Zhang et al., 2012 ▶; Rosa et al., 2018 ▶). 

## Discussion

 According to the presented results, glial cells have a dual role, causing inflammation and neurotoxicity or recovery and increasing the neuronal survival. In this way, effective control of the function of glial cells can be considered one of the most important therapeutic approaches in stroke. RSV as an anti-inflammatory and antioxidant, by modulating glial signaling pathways can lead not only to a decrease in the secretion of inflammatory cytokines and ROS generation but also to an increase in anti-inflammatory cytokines and growth factors of the glia, which ultimately prevents neurotoxicity, and apoptosis followed by increasing the viability of the neurons. However, our understanding of the precise mechanisms of RSV involved in glia activation after stroke is very limited and further studies are needed to understand these mechanisms in detail. Understanding these mechanisms is crucial for developing new therapeutic strategies for treatment of cerebrovascular disease.
